# Perception Versus Reality: The Use of Teach Back by Medical Residents

**DOI:** 10.3928/24748307-20190501-01

**Published:** 2019-07-01

**Authors:** Iris Feinberg, Michelle M. Ogrodnick, Robert C. Hendrick, Kimberly Bates, Kevin Johnson, Bingyan Wang

## Abstract

**Background:**

Health care providers (HCPs) may ask patients if they understand their diagnosis or instructions during clinic visits; patients often simply say yes. However, many patients leave with little idea of their medication and discharge instructions. Teach Back (TB) is a patient-centered health-literate technique that allows HCPs to confirm patient understanding during clinic visits.

**Objective:**

The purpose of this pilot study was to determine a relationship between perception and actual use of TB by medical residents in primary care outpatient clinics (providers, *N* = 16; clinic visits, *N* = 80) and, if the observed rate of TB was discordant with perception, did a TB skills training intervention have any impact on use of TB (clinic visit, *N* = 78). We were also interested in language used during TB and if use of TB was related to patient demographics or health literacy level.

**Methods:**

Medical residents' perception was measured using the “Always Use Teach-Back Confidence and Conviction Scale” (*N* = 16). Clinic visits were audiotaped and scored for use of TB (pre-intervention, *N* = 80; post-intervention, *N* =78). The intervention was a 1-hour TB skills training course. Content analysis was performed to understand the use of TB language.

**Key Results:**

Despite the high level of confidence/conviction about TB (*r*[16] = .669, *p* <. 05) TB was only used twice out of 80 visits during pre-intervention clinic visits. During post-intervention, use of TB increased to 41 times by 10 residents (c2[1, *N* = 16] = 6.533, *p* <. 05). TB language after the intervention was more collaborative; there was a relationship between gender and use of TB.

**Conclusion:**

Results from our pilot study identified three important observations that may be critical to improving health-literate physician communication: residents believe they are using TB in the clinic for many patients; use of TB was discordantly low at 2.5%; and a single 1-hour skills training intervention dramatically increased TB use to 53%. Residents used patient-centered TB language after the training intervention. **[*HLRP: Health Literacy Research and Practice*. 2019;3(2):e117–e126.]**

**Plain Language Summary:**

Medical residents believe they are using Teach Back to confirm patient understanding in the clinic 60% of the time when they actually used Teach Back only 2.5% of the time. After an educational intervention, they used Teach Back 53% of the time; Teach-Back language was collaborative and patient-centered, and all but two patients confirmed their medication and discharge plan.

The most common way patients receive health information is in verbal exchanges with health care providers (HCPs), yet up to 80% of this information is immediately forgotten, and almost one-half of what patients recall is incorrect (Kessels, 2003; McCarthy et al., 2012). How much and what patients remember and understand may be dependent on how skilled the physician is in delivering that information (Bodenheimer, 2018; Richard, Glaser, & Lussier, 2017; Samuels-Kalow, Hardy, Rhodes, & Mollen, 2016). When physicians receive training in health literate communication techniques, patient adherence in ambulatory settings is 1.62 times higher (Zolnierek & DiMatteo, 2009). In addition to other elements of verbal and nonverbal communication, including empathy and concern, HCPs share scientific, evidence-based information that affects patient adherence to medication and discharge instructions; however, research shows that less than 40% of people said their physician explained this information clearly (Alston et al., 2012).

Many physicians have little opportunity for feedback on their communication skills, and most have minimal formal communication training (Safeer & Keenan, 2005). Physician communication is a significant factor to patient understanding and safety (Lorincz et al., 2011); yet, not only is there a lack of medication counseling in outpatient medical visits, but physicians also overestimate their communication skills in both task-oriented and psychosocial communication (Antimisiaris & Cutler, 2017; Richard & Lussier, 2006; Tarn et al., 2006; Zolnierek & DiMatteo, 2009). Other barriers to patient-centered and health-literate communication skills include lack of time in medical education training, inadequate curricula, clinical instructors and supervising physicians who believe that communication skills cannot be learned, and lack of good role models in clinical practice (Ha & Longnecker, 2010; Heaven, Clegg, & Maguire, 2006; LeBlanc, 2015). Teaching HCPs how to use evidence-based communication tools presents an opportunity to improve clinical practice and patient outcomes by incorporating health-literate techniques that are accurate, complete, and understood by the patient (The Joint Commission, 2016).

Although some believe that health-literate communication skills are innate, subjective, and cannot be taught, data show that HCPs can learn good communication skills through systematic and intentional training (Howard, Jacobson, & Kripliani, 2013; M. F. Nogueira-Martins, Nogueira-Martins, & Turato, 2006; Rees, Sheard, & McPherson, 2002). According to the Association of American Medical Colleges, there are 19,500 new residents who graduate every year from 150 accredited US medical schools (American Association of Medical Colleges, 2018); communication skills are rarely taught as a separate curricular component but may be integrated into classroom and practicum experiences, and are often presented as the need to be empathetic and use plain language (Koh, Gracia, & Alvarez, 2014). Physicians are trained to collect subjective and objective patient information to make assessments and treatment plans, yet they are not trained in critical health-literate communication skills that can affect how information is shared, understood, and acted upon (Harper, Cook, & Makoul, 2007; Street, Gordon, Ward, Krupat, & Kravitz, 2005). In the United States, the Liaison Committee on Medical Education is responsible for accrediting medical school programs that lead to the “MD” degree; the American Osteopathic Association's Commission on Osteopathic College Accreditation accredits programs that lead to the “DO” degree. Both organizations require communication skills training as part of the core competencies, although the description is minimal; neither organization includes health literacy as a core competency (Liaison Committee on Medical Education, 2016).

Going from medical school into residency frequently leads to a decline rather than an increase in communication skills (Levinson & Pizzo, 2011). This decline could be due to residents receiving less feedback about communication skills when working directly with patients (Levinson & Pizzo, 2011). Further, medical students, physician assistant students, and internal medicine residents have reported low levels of confidence when working with patients who have low health literacy (Coleman, Nguyen, Garvin, Sou, & Carney, 2016). There are some communication techniques that medical students and providers can learn and incorporate into their routine conversations with patients to alleviate some of the adverse effects associated with low health literacy and reduce confusion about medication and discharge instructions (Bodenheimer & Grumbach, 2012; Coleman et al., 2016).

Physicians often believe that they have excellent communication and health literacy skills, but in actuality, this is not the case (Arnold, Coran & Koropeckyj-Cox, 2016; Morgan, 2013). In particular, most medical residents think they know how to communicate, yet they have had little instruction or practice (Ha & Longnecker, 2010; LeBlanc, 2015; Silverman, Kurtz, & Draper, 2005). Medical communication is vastly different than merely having a conversation due to varying patient levels of anxiety, emotions, asymmetrical power relationships, and health risks (LeBlanc, 2015; M. F. Nogueira-Martins et al., 2006; Silverman et al., 2005). The American Board of Medical Specialties and the Accreditation Council on Graduate Medical Education list “Interpersonal and Communication Skills” as 1 of 6 core competencies for residency training (NEJM Knowledge+, 2019); the communication sub-competency focuses on effective patient communication and shared decision-making (Holmboe, Edgar, & Hamstra, 2016; Liaison Committee on Medical Education, 2016). Evidence shows that health-literate communication skills can be taught, learned, and used by medical residents and practicing clinicians (Coleman et al., 2016; Fallowfield et al., 2002; Heaven, Clegg, & Maguire, 2006; Rees et al., 2002; Yedidia et al., 2003) with the Institute of Medicine calling for structured communication skills assessment as part of continuing medical education and physician licensing (Kindig, Panzer, & Nielsen-Bohlman, 2004).

Teach Back (TB) is an evidence-based communication tool that allows physicians to confirm patients understand what was said during the verbal exchange. HCPs engage the patient in a conversation by asking the patient to explain what the HCP said in his or her own words; this allows the HCP to verify if the patient understands, reinforce essential points, and correct anything that might not be right (Klingbeil & Gibson, 2018; MacLean, Kelly, Geddes, & Della, 2018; Slater, Dalawari, & Huang, 2013). It is vital that the HCP takes responsibility for what they are communicating so that the patient does not feel that he or she is being tested; phrases like “I gave you a lot of information today” and “I want to make sure I was clear” are indications of patient-centered and collaborative TB language (Bodenheimer, 2018; Klingbeil & Gibson, 2018). Data show that even a brief TB educational intervention for multidisciplinary HCPs can improve quality of care and patient safety by correcting patient misunderstandings and clarifying information (Klingbeil & Gibson, 2018). Evidence supports the use of TB as a method for enhancing communication, reducing the cognitive load on patients and their families, and improving patient safety (Berry et al., 2014; Bodenheimer, 2018; Howard et al., 2013).

The purpose of this mixed-methods pilot study was to determine if there was a relationship between the perception of using TB and the actual use of TB during clinic visits for first, second, and third year family and internal medicine residents; that is, if the medical residents' health literacy skills matched their health literacy practice with patients. If the observed rate of TB was discordant with the residents' perception, we wanted to know if TB would be used more frequently in the clinic after a skills intervention. We were also interested to know what language residents used and how patients responded to the use of TB, and if that was characterized by patient demographics or health literacy levels with the following research questions:

1. Is there a relationship between how physicians perceive they are using TB and actual use of TB during patient clinic visits?
2. Is there a difference in physician use of TB during patient clinic visits before and after a TB intervention?
3. What TB language is used by residents pre- and post-intervention and how do patients respond?
4. Is the use of TB by residents informed by patient demographics or health literacy levels?

## Methods

### Sample

We recruited first, second, and third year family medicine and internal medicine residents who provide patient care in two clinics affiliated with a regional hospital based in suburban Atlanta, GA, and who see adult patients on multiple weekdays. Residents were introduced to the study and recruited at a regularly scheduled graduate medical education meeting. Supervisors and instructors recused themselves from that meeting. The research team informed residents that we would audiotape five of their patient clinic visits before a skills training intervention and five patient clinic visits after the intervention. Patient recruitment took place in the waiting areas of the internal medicine and family medicine clinics prior to their visit with the residents; all were age 18 years and older and spoke English as their primary language. They were consented to audiotape their visit with the physician; a guest or spouse accompanying the patient consented as well if they joined the patient in the examination room. We received Institutional Review Board approval for this study from both Georgia State University and Gwinnett Medical Center.

### Measures

The medical residents completed a demographic survey that included program of study, year of residency, gender, age, race/ethnicity, country of birth, and native language. They also completed the “Always Use Teach-Back Conviction and Confidence Scale” (Abrams et al., 2012). This five-question tool measures HCP belief in communication skills, conviction in the importance of using TB in the clinic, and perceived use of TB in the clinic (**Table 1**). The Conviction and Confidence Scale is a widely used assessment tool developed in 2011 based on the stages of change, behavior change, and the motivational interviewing literature of Prochaska, DiClemente, and Rollnik (M. A. Abrams, personal communication, October 25, 2018).

For the patients, we collected demographic information (age, gender, race/ethnicity, native language) and assessed their health literacy level using the Newest Vital Sign (NVS) (Weiss et al., 2005). The NVS is an ice-cream container nutrition label with six questions that are read to the participant and is designed to simply and quickly estimate a person's health literacy level. The test takes 3 minutes to complete; fewer than four correct answers indicates the likelihood of limited HL (**Table 2**).

Once the patient entered the examination room, a researcher placed a tape recorder on the counter in the room. The researcher did not stay in the examination room. Audiotapes were transcribed by three graduate research assistants; the research coordinator coded the transcripts according to a coding rubric (**Table 3**). Any spouse/guest comments were deleted prior to coding; our focus was on the use of TB by the resident and the patient's response to TB. The principal investigator (PI) (I. F.) reviewed all the transcripts; any conflicts were discussed and resolved between the research coordinator and the PI.

The PI skills training intervention was a 1-hour training session on TB and took place during a regularly scheduled half-day education session for the medical residents; the intervention occurred after completion of the first phase of data collection (*N* = 80, audiotapes). All residents, whether part of the study or not, attended the skills training intervention session. The PI presented a 1-hour PowerPoint training session that included discussion of the first-phase results, the importance of TB, videos of TB, and examples of TB language to use. This presentation is modeled after the “Always Use Teach-Back” training tool-kit, which includes lectures, demonstration, and videos (Abrams et al., 2012). We included time to address concerns and questions voiced by the residents that included the amount of additional time using TB might take, what to do if a patient is unable to teach back the information, and how to address incorrect responses from the patients. An additional 78 patients were audiotaped during the 3-month period after the TB intervention.

## Results

More than one-half of the 16 medical residents were male and White; average age was 28.3 years. Ten (63%) were in the family medicine program; of the 16 residents, 50% were in year 1 of their postgraduate residency. Close to 70% were born in the United States. The 16 medical residents saw a total of 158 patients (10 per resident except one resident had 8 patients in post-intervention). One-half of the patients were between ages 45 and 64 years (50%), and one-half (51%) were African American; 60% were female, and almost all had a high school diploma or equivalency degree (96%). Less than one-half (45%) had adequate health literacy as measured by the NVS. Demographic details are shown in **Table 4**.

To answer Research Question 1, (Is there a relationship between how residents perceive they are using TB and actual use of TB during patient clinic visits?), we compared responses to the Confidence and Conviction Scale to actual use of TB during audiotaped office visits pre-intervention. There are two sections to the Confidence and Conviction Scale: a 10-point Likert scale is used to gather information about the conviction of the importance and use of TB and the perceived communication ability of the resident and a 4-point Likert scale (1 = *never*, 2 = *some*, 3 = *many*, 4 = *all*) was used to record the perceived actions of the resident during the clinic visit. The 10-point and 4-point Likert scales are ordinal and were analyzed using parametric procedures (Carifio & Perla, 2008; Norman, 2010). Using Pearson correlation, the means show that the items “How convinced are you that it is important to use Teach Back?” and “Rate the importance of explaining and checking again if the patient is unable to explain their care plan” are correlated at .669, *p* = .034, indicating agreement among the residents that TB and other strategies like TB are important (indicated mean levels of 8.3 and 9 on a 10-point scale). Analysis of audio-taped office visits showed a discordance between perception of using TB (answering “for many patient visits” on the Confidence and Conviction Scale) and actually using TB (audiotaped analysis shows it was used for only two patient visits); the perception was 60%, and actual use was 2.5%. SPSS 24 was used for all frequencies and correlations.

To answer Research Question 2, (Is there a difference in resident use of TB during patient clinic visits before and after a TB intervention?), the frequency of TB was measured by analysis of audiotaped clinic visits both prior to and after the intervention. The actual use of TB increased substantially from 2 reported uses pre-intervention to 41 uses post-intervention. A 2 × 2 contingency table examined the 16 participants regarding the proportion of residents that used TB pre-intervention compared to the proportion post-intervention (**Table 5**). For the pre-intervention proportion, 2 of 16 residents used TB; for the post-intervention proportion it was 10 of 16. Using Yates correction for small sample sizes, the Chi-Squared value is 6.533 with 1 degree of freedom (*p* = .011), which indicates a statistically significant increase. Thus, there was a strong and positive impact in use of TB after a skills training intervention. All analyses were conducted using SPSS 24.

To answer Research Question 3, (What TB language is used by residents pre- and post-intervention and how do patients respond?), the language used for the two instances of TB pre-intervention was focused on testing the patient's knowledge and the resident took little responsibility for his or her role in the communication. In both instances the questions were about medication use, and the patient responded by correctly repeating the medication instructions:

Resident (pre-example 1): Can you tell me again what I just told you?

Patient: You want me to take the other medication, the Tylenol.

Resident: So, when you have a very mild headache, when the headache starts, what should you do?

Patient: When it starts, I should take it that, when its severe take the Tylenol. When it doesn't help me, you know, I should take it like 3 times, the Tylenol. When it doesn't help me then I should take the other medication.

Resident (pre-example 2): Okay (chuckles) alright and what about the prescription, Imitrex, what did I say about that?

Patient: It's to help with my headaches.

Resident: How did I say take it?

Patient: Once at onset and if that, and I can do it again in 2 to 3 hours later.

Resident: Mmm… and how many pills are you not supposed to…

Patient: Not more than two a day.

The language used throughout the post-intervention TB instances was more collaborative than pre-intervention. The most frequently used phrases in the 43 post-intervention visits were “so I know we talked a lot about” (16% of the time) and “so I know you understand/just to make sure you understand/can you tell what you understand” (26% of the time). In the post-intervention phase, the resident took responsibility for his or her communication by prefacing direct questions with phrases such as:

“So I know we talked about a lot but can you basically tell me what the plan is?”

“So can you summarize for me kinda what we talked about so that I know you understand what we talked about? What are we going to do for the shoulder”?

“So if you can real quick maybe two or three things that we talked about and what we are going to do going forward.”

“Okay so I know we talked about a lot of stuff today. … I just want to make sure you kinda understood everything so kinda could you reiterate everything we talked about?”

In all instances except two, patients responded by explaining back what the resident said in their own words. In the two instances where the patient did not explain back, they responded with “I'm good” and “Everything is good.”

To answer Research Question 4, (Is the use of TB by residents informed by patient demographics or health literacy levels?), we ran independent *t* tests for patients dependent on whether they received TB. There was no significant difference between use of TB for age, race/ethnicity, native language, or health literacy level. There was a significant difference in the use of TB for men (*M* = .37, *SD* [standard deviation] = .49) versus for women (*M* = .21, *SD* = .41), *t*(156) = 2.16, *p* = .02.

## Discussion

Results from our pilot study identified three important observations that may be critical to improving physician communication skills: (1) family medicine and internal medicine residents are convinced that TB is important and that they are using it with patients 60% of the time; (2) these same residents use of TB was discordantly low at 2.5%; and (3) a single 1-hour skills training intervention dramatically increased TB use to 53%. We further identified that medical residents use more collaborative TB language and use it more often after a brief skills intervention and that only gender had a relationship with use of TB.

The use of health-literate communication techniques like TB should be universal, i.e., it should be used for all patients regardless of health literacy level (Liang & Brach, 2017). Health literacy is contextual and situational, that is, anyone can have low health literacy based on what is happening now; thus, health providers should treat all patients as if they have low health literacy (Liang & Brach, 2017). TB is an important universal tool to confirm patient understanding. Although there has been some improvement in the use of TB, only 29% of patients report that their physician asked them to explain what they understood about their care instructions (Liang & Brach, 2017). Understanding medication and discharge instructions are crucial factors to therapy adherence, health outcomes, and patient safety (Engel et al., 2009; Hawk & Evans, 2013; Tamura-Lis, 2013). In our study, medical residents had strong positive beliefs about the value of using TB with their patients both before and after a skills training intervention, yet they did not actually use TB until after a focused skills training.

Our findings show that specific communication skills like TB can be learned by medical residents, and when learned, can be used; occurrences of TB increased from 2.5% before a training intervention to 53% afterwards. We also learned that there is a range of how medical residents perform TB from those who do it all the time to those that do it some of the time to those that don't do it all. In addition to our results, there is extant strong evidence that effective clinical communication skills can be learned by HCPs (Denniston, Molloy, Nestel, Woodward-Kron, & Keating, 2017; Yedidia et al., 2003).

We show that even a simple 1-hour skills training using lecture, videos, and discussion may have an impact on what medical residents learn and enact. A particularly important part of the training was discussion with the medical residents where they shared concerns about using TB; a key concern was figuring out exactly which words to use. Through discussion and practice with the expert trainer, residents learned several different phrases they could use as well as what to do when the patient struggles with teaching back the information. All residents who used post-intervention TB were able to use one of the phrases we discussed or something similar that may have been easier for them to say. All of the patients except two were able to correctly respond; thus, residents did not have to deal with any patient who did not understand and could not confirm their medication or discharge instructions. We determined that there was not a significant difference between the length of clinic visit with use of TB. Although we did not evaluate the difference between a multihour/day/course intervention and our 1-hour intervention, our results show that a significantly greater number of residents used TB post-intervention (10 versus 2 pre-intervention), thus leading us to believe that an expertly led skills training intervention with time for discussion was successful in this setting.

Based on these findings, we are beginning a research study on adding TB as a curricular component for first-year medical students and will track their use of TB in the simulation laboratory with patient actors through the end of their second year. The students will be in the simulation laboratory at least 3 times each semester; we will evaluate their use of TB after each semester to determine if additional training is needed or if a one-time training session is adequate. In addition, we are working with respiratory therapy students and second-year nursing students to assess if a TB curricular component is effective in their simulation laboratory experiences as well. We will be interviewing students to better understand facilitators and barriers to use of TB. Future research should include a longitudinal evaluation of TB use after training, either within medical education or during postgraduate training such as in a residency program. We are also interested in knowing if patients' recall a few days postclinic visit is improved if the HCP uses TB during the patient encounter.

## Study Limitations

Our study had a small number of medical resident participants (*N* = 16) so the power is rather low (.46) with a moderate effect size, although small samples are not unusual for pilot studies such as this. A second limitation is the short follow-up time between a skills training intervention and assessing TB use, as our study accrued only five patients per resident post-intervention and we accomplished this within 3 months. We also did not know if medical residents had been exposed to TB training in either medical school or in their postgraduate training years prior to our study. Finally, we did not measure if a longer TB intervention (i.e., multiple exposures to TB in training) would have a greater effect on medical residents than a one-time skills training course.

## Figures and Tables

**Table 1:** Conviction and Confidence Scale

**Question**	**Scale**
How would you rate your communication skills with patients?	1 (*poor*) to 10 (*excellent*)
How convinced are you that it is important to use Teach Back (ask patients to explain key information in their own words)?	1 (*not at all important*) to 10 (*very important*)
How confident are you in your ability to communicate with patients from all backgrounds, cultures, and languages?	1 (*not at all confident*) to 10 (*very confident*)
How often do you ask patients to explain what they need to know or do to take care of themselves?	1 (*never*); 2 (*some patient visits*); 3 (*many patient visits*); 4 (*all patient visits*)
How would you rate these elements of communication with patients? Use a caring tone of voice and attitude Use plain language Ask the patient to explain, in his or her own words, what they were told Use open-ended questions Avoid asking questions that can be answered with a yes or no Take responsibility for making sure you were clear Explain and check again to see if the patient is unable to explain his or her care plan Use reader-friendly print materials to support learning Document use of and patient's response to his or her care plan Include family members/caregivers if they are present Provide a written care plan	1 (*not at all important*) to 10 (*very important*)

**Table 2:** Newest Vital Sign Health Literacy Test

**Question**	**Correct Answer**
If you eat the entire container, how many calories will you eat?	1,000
If you are allowed to eat 60 g of carbohydrates as a snack, how much ice cream could you have?	Any amount up to 1 cup, half the container, or 2 servings
Your doctor advises you to reduce the amount of saturated fat in your diet. You usually have 42 grams of saturated fat each day that includes one serving of ice cream. If you stop eating ice cream, how many grams of saturated fat would you be consuming each day?	33
If you usually eat 2,500 calories in a day, what percentage of your daily value of calories will you be eating if you eat one serving of ice cream?	10%
Pretend that you are allergic to the following substances: penicillin, peanuts, latex gloves, and bee stings. Is it safe for you to eat this ice cream? (If they answer no to previous question) Why not?	No It has peanut oil

**Table 3:** Transcription Coding Rubric for Teach Back (Examples)

I gave you a lot of information and I want to make sure I was clear—what are you supposed to do to take care of *X*? What will you tell your spouse about today's visit? Can you repeat what I told you about your medicine? We've talked about a lot of things. What is our plan for treating your *X*? Can you summarize what I've told you? What else are you going to do the next time? If someone asks you what would you be doing until we follow up again, what would you tell them? Tell me how you understand our plan. So, for the *X*, can you tell me what you're going to do?

**Table 4:** Demographic Characteristics of Medical Residents (*N* = 16) and Patients (*N* = 158)

**Characteristic**	***n* (%)**

Residents	

Gender	
Male	10 (63)
Female	6 (37)

Race/ethnicity	
White	9 (56)
Asian	3 (19)
African American	2 (13)
Hispanic	1 (7)
Other	1 (7)

U.S. born	
Yes	11 (69)
No	5 (31)

Postgraduate program	
Family medicine	10 (63)
Internal medicine	6 (37)

Postgraduate year	
First	8 (50)
Second	5 (31)
Third	3 (19)

Patients	

Gender	
Female	95 (60)
Male	63 (40)

Age (years)	
18–24	13 (8)
25–34	14 (9)
35–44	30 (19)
45–54	45 (28)
55–64	34 (22)
65+	22 (14)

Race/ethnicity	
African American	81 (51)
White	51 (32)
Hispanic	13 (9)
Asian	2 (1)
Other	11 (7)

High school diploma or equivalency	
Yes	151 (96)
No	7 (4)

Health literacy score	
0–1 High likelihood of low health literacy	40 (25)
2–3 Possibility of limited health literacy	47 (30)
4–6 Adequate health literacy	71 (45)

**Table 5:** Cross-Tabulation Use of Teach Back by Medical Residents Pre- and Post-Intervention

**Use of Teach Back**	**Did Not Use Teach Back**	**Did Use Teach Back**	**Total**
Pre-intervention	14	2	16
Post-intervention	6	10	16
Total	20	12	32
